# Care of Burns in Scotland: 3-year data from the Managed Clinical Network National Registry

**DOI:** 10.1186/cc13266

**Published:** 2014-03-17

**Authors:** CJ Gilhooly, J Kinsella

**Affiliations:** 1 Glasgow Royal Infirmary, Glasgow, UK; 2 University of Glasgow, UK

## Introduction

The Managed Clinical Network for Care of Burns in Scotland (COBIS) was launched in April 2007. Primary aims included establishing and maintaining a registry of complex burn injury in Scotland and setting mechanisms to regularly audit outcome of burn treatment against nationally agreed standards of care. On behalf of COBIS, we present 3-year incidence and mortality data of Scottish patients admitted with a complex burn injury in this abstract.

## Methods

From January 2010 onwards, data were prospectively collected for all patients in Scotland with complex burn injury admitted to Scottish burns units. Data collection was initially on a paper *pro forma, *but subsequently evolved into a web-based audit data capture system to securely link hospital sites involved in the delivery of care of complex burns. Data collected included extent and mechanism of burn, presence of airway burn or smoke inhalational injury, comorbidities, complications, length of stay, interventions and mortality. Quality, completeness and consistency of data collection are audited with feedback to the individual units.

## Results

In a population of approximately 5.3 million, the annual incidence of complex burn injury is 499 to 537 (9 to 10 per 100,000). The incidence of a major burn is 5% of burn admissions. The hospital mortality from a burn is 1 to 2.2%. See Table 1.

**Table 1:** Numbers of complex burns in Scotland 2010 to 2012

	2010	2011	2012
Adult	304	392	399
Paediatric	195	185	138
Adult >15%	27	28	26
Paediatric >15%	0	2	4
Mortality	5	7	13

## Conclusion

From these data, Scotland now has comprehensive national figures for complex burn injury. This allows for benchmarking against other international indices, few of which provide comprehensive data. COBIS data can now also be correlated with other mortality data sources. As data quality improves, detailed analysis of mortality data will allow COBIS to identify contributing issues affecting burns patients. Some issues identified already are that patients with burns often die soon after their discharge from hospital of other related and unrelated causes. Subsequent analysis of this will allow COBIS to identify and address issues that may be contributing to these statistics.

